# Prognostic limitations of the Eurotransplant-donor risk index in liver transplantation

**DOI:** 10.1186/1477-5751-12-18

**Published:** 2013-12-24

**Authors:** Benedikt Reichert, Alexander Kaltenborn, Alon Goldis, Harald Schrem

**Affiliations:** 1 Department of General, Visceral and Transplant Surgery, Hannover Medical School, Carl-Neuberg Str. 1, 30625, Hannover, Germany; 2 General and Thoracic Surgery, Universitätsklinikum Schleswig Holstein, Kiel, Germany; 3 Federal Armed Forces Medical Center Hannover, Hannover, Germany; 4 Lean Six Sigma Black Belt, LM Advisors, Amstelveen, Netherlands

**Keywords:** Prognostic model, Accuracy, Goodness-of-fit, Score, Prediction, Validation

## Abstract

**Background:**

Liver transplantation is the only life-saving therapeutic option for end-stage liver disease. Progressive donor organ shortage and declining donor organ quality justify the evaluation of the leverage of the Donor-Risk-Index, which was recently adjusted to the Eurotransplant community’s requirements (ET-DRI). We analysed the prognostic value of the ET-DRI for the prediction of outcome after liver transplantation in our center within the Eurotransplant community.

**Results:**

291 consecutive adult liver transplants were analysed in a single centre study with ongoing data collection. Determination of the area under the receiver operating characteristic curve (AUROC) was performed to calculate the sensitivity, specificity, and overall correctness of the Eurotransplant-Donor-Risk-Index (ET-DRI) for the prediction of 3-month and 1-year mortality, as well as 3-month and 1-year graft survival. Cut-off values were determined with the best Youden-index. The ET-DRI is unable to predict 3-month mortality (AUROC: 0.477) and 3-month graft survival (AUROC: 0.524) with acceptable sensitivity, specificity and overall correctness (54% and 56.3%, respectively). Logistic regression confirmed this finding (p = 0.573 and p = 0.163, respectively). Determined cut-off values of the ET-DRI for these predictions had no significant influence on long-term patient and graft survival (p = 0.230 and p = 0.083, respectively; Kaplan-Meier analysis with Log-Rank test).

**Conclusions:**

The ET-DRI should not be used for donor organ allocation policies without further evaluation, e.g. in combination with relevant recipient variables. Robust and objective prognostic scores for donor organ allocation purposes are desperately needed to balance equity and utility in donor organ allocation.

## Background

Expansion of the organ donor pool by using grafts with reduced quality has become reality in transplant centers around the world due to increasing donor organ shortage and raising numbers of patients on the waiting lists. This has led to an increased awareness of the associated risks for patient and graft survival [1-16]. Thus, a series of attempts to develop objective scores for the assessment of donor liver quality with significant influence on patient and/or graft survival have been made [1,2,4-9]. In the transplant literature there is a current debate on the criteria for statistical validation of prognostic scores in liver transplantation that may be relevant for fair and just allocation rules that balance equity and utility [10,12,13,17,18]. Astonishingly, there is still a high heterogeneity of statistical approaches to the validation of prognostic scores even though Jacob et al. published as early as 2005 uniform quality criteria for the design, validation and reporting of prognostic scores in liver transplantation more than eight years ago [18]. Last year we have reported from our centre that the Donor-Risk-Index is not applicable to our patients for the prediction of three month mortality, three month patient survival, three month graft survival as well as the necessity of acute retransplantation within thirty days [13]. In contrast Blok et al. claimed in the same year that they were able to validate the value of the DRI for the prediction of three month, one year and three years graft survival in the Eurotransplant region [3]. In this study as well as in earlier studies the authors could not demonstrate areas under the receiver operating characteristic curve (AUROC) greater than 0.700 for these predictions [3,4,7,13]. This raises the fundamental question: What are the criteria for a robustly validated prognostic model?

A relevant body of scientific literature in the bio-statistical world agrees on the outstanding value of the receiver operating curve analysis for the determination of the value of clinical prognostic scores [18-22]. Therefore, we present in this paper a validation study on the recently published Eurotransplant-Donor-Risk-Index (ET-DRI) and evaluate its value for prognostic decision making in the cohort of our center.

This study is a timely contribution to the urgently needed debate on prognostic models in liver transplantation with potential impact on donor organ allocation policies. The recent liver transplant scandals in Germany revolve around waiting list manipulations that affected donor organ allocation directly and undermined public trust in current donor organ policies [23].

## Results

### ET-DRI score

The variables’ frequencies, range and percentages used in the ET-DRI of our study population are summarized in Table 1. The mean ET-DRI in this cohort was 1.79 (median 1.70, range: 1.14 - 3.79).

**Table 1:** Shown are the donor characteristics of our study population’s variables which are part of the ET-DRI model

**Variables**		
	**n (%)**	
COD anoxia	24 (8.2%)	
COD cerebrovascular accident	181 (62.1%)	
COD other	86 (29.6%)	
DCD	nil	
Split LTX (yes/no)	20 (6.9%)	
regional share (yes/no)	253 (86.9%)	
national share (yes/no)	38 (13.1%)	
rescue offer (yes/no)	53 (18.2%)	
age group <40 years	71 (24.4%)	
40 ≤ age < 50 (in years)	69 (23.7%)	
50 ≤ age < 60 (in years)	105 (36.1%)	
60 ≤ age < 70 (in years)	52 (17.9%)	
70 ≤ age (in years)	12 (4.1%)	
	**mean (median)**	**range**
CIT in h	9.75 (9.6)	2.4 – 27.3
latest labGGT (U/L)	70.66 (43.0)	5 – 775
Age in years	48.4 (51.0)	12 – 74

(COD = cause of death, DCD = donation after cardiac death, LTX = liver transplantation, CIT = cold ischemic time).

Major results of the analyses of the ET-DRI’s predictive capabilities for the study endpoints are summarized in Table 2.

**Table 2:** Shown are the results of the analyses of the study endpoints

**Endpoint**				**ET-DRI cut off values**			
	**AUROC**	**95%-CI**	**Logistic regression p-value**		**Sensitivity**	**Specificity**	**Overall correctness**
**3-month mortality**	0.477	0.390-0.564	p = 0.692	2.06	26.7%	81.4%	54%
**1-year mortality**	0.492	0.405-0.579	p = 0.573	2.06	26.7%	81.4%	54%
**3-month graft survival**	0.524	0.477-0.601	p = 0.475	1.95	38%	74.5%	56.3%
**1-year graft survival**	0.540	0.473-0.607	p = 0.475	1.84	47.4%	63.6%	55.5%

AUROC = area under the receiver operating characteristic curve; 95%-CI = 95%-Confidence Interval

### Prediction of 3-month mortality

**Figure 1:**
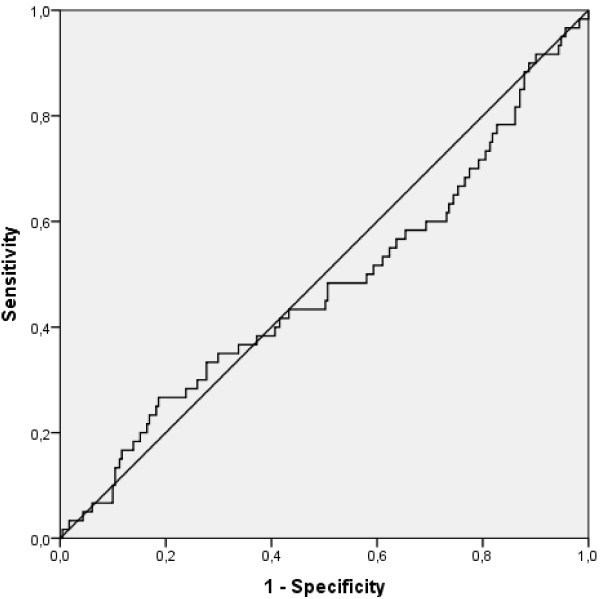
Shown is the ROC-Curve for the prediction of 3-month mortality after liver transplantation with the ET-DRI (AUROC = 0.477; 95%CI: 0.390-0.564).

**Figure 2:**
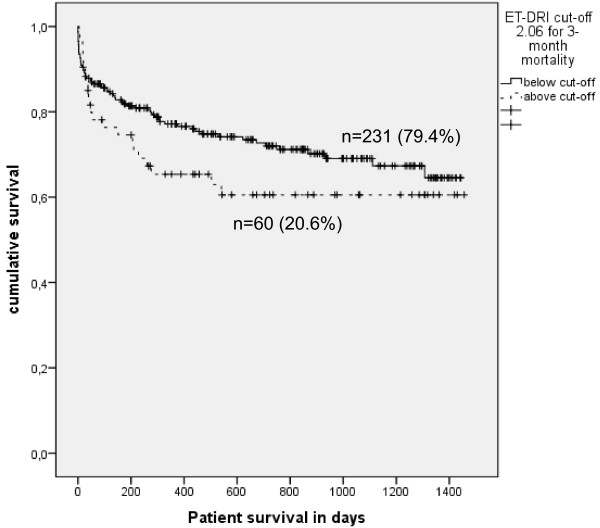
**Kaplan Meier survival analysis above and below the ET-DRI cut-off values for the prediction of 3-month mortality (2.06) shows that this cut-off value has no significant influence on long-term survival (p = 0.172; Log Rank).** The curve clearly demonstrates that the ET-DRI has no influence on long-term survival and limited influence on survival within the first 90 days.

With an AUROC of 0.477 (95%-CI: 0.390-0.564) the ET-DRI failed to predict 3-month mortality after liver transplantation in our study population (see Figure 1). This result could be confirmed by binary logistic regression (p = 0.692). The Hosmer-Lemeshow goodness of fit test did show that the model fit of the logistic regression model was adequate (p = 0.542). Kaplan-Meier survival analysis above versus below the calculated cut-off level of the ET-DRI for the prediction of 3-month mortality (2.06) did not reach statistical significance (p = 0.172, Log Rank; see Figure 2).

### Prediction of 1-year mortality

Further, the ET-DRI did not reach a relevant AUROC for the prediction of 1-year mortality (AUROC 0.492) (see Table 2). A well fitted binary logistic regression model (p = 0.262, Hosmer-Lemeshow test) revealed no statistically significant influence of the ET-DRI values on 1-year mortality (p = 0.573). The calculated cut-off level for this prediction is identical to the cut off for the prediction of 3-month mortality. Therefore, it is no surprise that the Kaplan-Meier survival analysis above versus below this cut-off level did also not reach statistical significance (p = 0.230, Log Rank; see Figure 2).

### Prediction of 3-month graft survival

**Figure 3:**
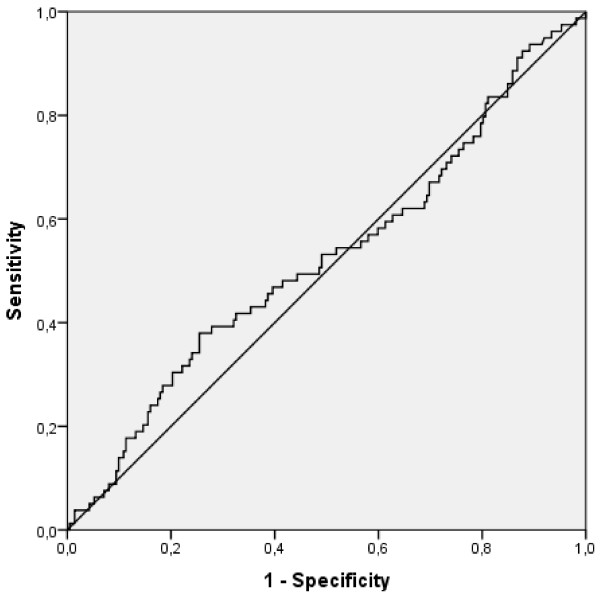
Shown is the ROC-Curve for the prediction of 3-month graft survival after liver transplantation with the ET-DRI (AUROC of 0.524 (95%CI: 0.447-0.601, SD 0.039).

**Figure 4:**
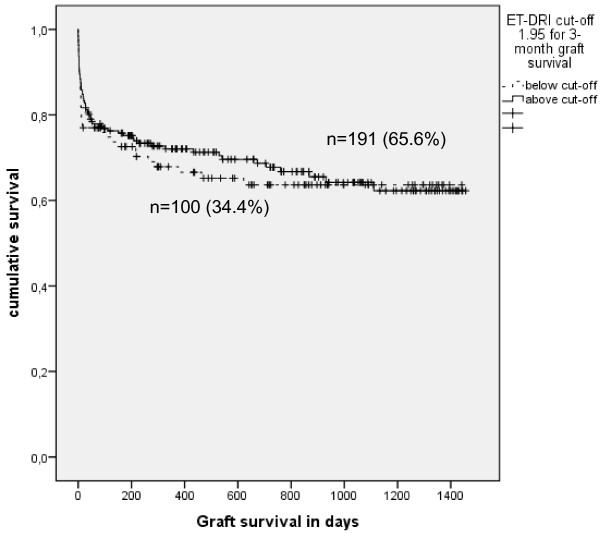
**Kaplan Meier survival analysis above and below the ET-DRI cut-off values for the prediction of 3-month graft survival (1.95) shows that this cut-off value has no significant influence on long-term graft survival (p = 0.655; Log Rank).** The curve clearly demonstrates that the ET-DRI has no influence on long-term graft survival.

With an AUROC of 0.524 (95%CI: 0.447-0.601) the ET-DRI failed to predict 3-month graft survival after liver transplantation in our study population (see Figure 3). Further, investigation of the ET-DRI as a risk factor for 3-month graft loss with a well fitted binary logistic regression model (p = 0.491, Hosmer-Lemeshow test) remained insignificant (p = 0.475). Kaplan-Meier analysis of graft survival above versus below the determined cut-off level for the prediction of 3-month graft survival did not reach statistical significance (p = 0.655, Log Rank; see Figure 4).

### Prediction of 1-year graft survival

Applied to our data the ET-DRI is not able to predict 1-year graft survival as shown by ROC-curve analysis results (AUROC = 0.540; 95%CI: 0.473-0.607). In binary logistic regression, which was performed with adequate model goodness of fit (p = 0.631, Hosmer-Lemeshow test), the ET-DRI was no statistically significant risk factor for graft loss within one year after transplantation (p = 0.163). Kaplan-Meier analysis of graft survival above versus below the determined cut-off level (1.84) did not reach statistical significance (p = 0.083, Log Rank).

### Power calculation and sample size

Since the focus of this paper is prognostic estimation rather than hypothesis testing, strictly speaking power calculations are not directly relevant. The rationale for a sufficient sample size in the presented study is the fact that all 95%-CIs of the AUROCs are sufficiently narrow to exclude 0.700, the nominal criterion value for utility [20].

## Discussion

The ET-DRI was originally developed with thousands of patients transplanted in the Eurotransplant region including many transplant centers and the authors claimed that the ET-DRI is of prognostic relevance for graft survival after liver transplantation [5]. We believe that the fundamental discrepancy to our results and interpretation can be related to the fact that the ET-DRI as shown by Braat et al. cannot reach a *c*-index above 0.700 for the prediction of graft survival in thousands of liver transplant recipients [5]. This finding could be confirmed in our study with AUROCs <0.700.

Braat et al. [5] used the previously published DRI [7] which was developed in the United States and they attempted to tailor and adapt the DRI to the donor population in the Eurotransplant region. The goal of our study was to test the ET-DRI as a prognostic model in our centre with current MELD-based liver allocation rules. Braat et al. used a dataset of recipients who were transplanted in the vast majority of cases outside MELD-based organ allocation between 2003 and 2007. MELD-based liver allocation was introduced in Germany in December 2006 [5]. Braat et al. claimed in their paper which was published in 2012 that the ET-DRI should be used for liver allocation in the future [5]. We therefore believe that our analysis of the ET-DRI with a current cohort is fully justified in order to assess the potential merit of the ET-DRI for the future. Braat and colleagues clearly stated quite righteously that Eurotransplant is divided into different regions; i.e. for Austria, Belgium/Luxembourg, Croatia, the Netherlands and Slovenia each country is considered as one region, whereas Germany is divided into seven regions. Therefore the term allocation is divided into local (transplant center is in the procurement area), regional (transplantation and procurement area within the same country, or region in Germany), and national or extra-regional (anywhere in Eurotransplant, but outside the region) [5]. Interestingly, the ET-DRI calculation formula as shown by Braat et al. does not contain a criterion for local share. Our analysis of the influence of local versus regional versus national share on transplant outcome did not show a significant impact of local share on outcomes in our cohort (p < 0.05; Chi^2^).

Currently, Avolio et al. have claimed that ROC-curve analysis is not the adequate measure to assess the value of prognostic models for graft or patient survival, especially if they include donor organ variables. Additionally, they postulated that it would be preferable to use time-dependent ROC-curve analysis [17]. This notion is an expansion of the current thinking as expressed in the majority of recent bio-statistical publications [18-21]. Sensitivity, specificity and area under the ROC-curve are often used to measure the ability of survival models to predict future risk [18-21]. Estimation of these parameters is complicated by the fact that these parameters are time-dependent. Furthermore, censoring affects their estimation just as it affects estimation of survival curves or coefficients of survival regression models [22]. In the presented study only relatively short time windows have been investigated. Therefore, application of time-dependent ROC-curve analysis is in our view not justified.

There is currently no objective score for the assessment of donor organ quality available that would be able to predict short-term patient and graft survival with acceptable overall correctness ((sensitivity + specificity)/2). It appears safe to assume that extended donor criteria organs with extreme properties would affect graft survival negatively in the short-term and lead to initial graft non-function which is known to be associated with increased patient mortality [1,2,4-9,14,15]. Current prognostic scores for donor organ quality are unable to demonstrate significant statistical influences on short-term graft survival as robust prognostic tools [1,2,4-10,12-15]. We believe that these currently available scores may have missed potentially critical parameters and/or combinations of donor organ variables and/or their interaction that may be critical for a robust prognostic score to predict short-term graft survival with acceptable overall correctness. The design of many prognostic scores in the medical literature, including the ET-DRI has failed so far to assess multivariable co-linearity of the influences of variables on the target output variable (e.g. graft survival) [5,18,26].

Hanley and McNeil have shown that *c* is identical to a widely used measure of diagnostic discrimination, the AUROC in ROC-curve analysis [20,21]. A value of *c* of 0.5 indicates random prediction and a value of 1 indicates perfect prediction. A model having *c* greater than roughly 0.7 has some utility in predicting the responses of individual subjects [21]. The *c*-index 0.624 which was published before for the prediction of graft survival with the ET-DRI in a large Eurotransplant cohort [5] appears therefore as a measure of limited utility of this score.

This study is limited by the relatively small number of enrolled patients (n = 291). However, none of the 95%CIs included a relevant AUROC >0.700. Moreover, organ procurement from donors after cardiac death is a relevant factor of the original population, in which the ET-DRI was created [5]. Unfortunately we were not able to include this specific donor group, because liver donation after cardiac death is not applied in Germany. Thus, the results of this analysis may be confounded by the absence of these cases.

Taken together we believe that the ET-DRI or any other donor risk score should not be used for donor organ allocation rules without adequate criticism of its methodological limitations. The transplant community desperately needs a debate on the power and limitations of prognostic scores in liver transplantation which should lead to a consensus. In our opinion Jacob et al. have made a reasonable proposal for the quality assessment of prognostic models for liver transplantation [18].

The application of a previously developed score to a new dataset premises that the characteristics of both reference and study populations should result in overlapping or at least should present similar patterns [17]. This expectation is met with our study population which is a subset of the Eurotransplant population which was used by Braat and colleagues [5].

We believe that the reasons for our findings which somehow contrast the previously published findings [5] are maybe due to a strong center bias. This notion is likely because our population constitutes a subpopulation of the one that was investigated by Braat et al. [5]. Further, the fact that at least two transplant centers within the Eurotransplant region are known to believe that rescue allocated organs do not affect outcome after of liver transplantation [27,28] and the fact that the percentage of accepted rescue allocated livers for transplantation differs widely within the Eurotransplant region may contribute to this center bias. In our center the percentage of rescue allocated livers was comparatively low (18.2%) while approximately 30% of livers in the Eurotransplant region are transplanted after rescue allocation [27].

Another critical aspect in donor organ assessment is the histological diagnostics. Not all donor livers are biopsied. Moreover, the frequency of biopsies is completely dependent on the indication to perform a histological examination. This subjective and irregular indication is the responsibility of the explant surgeon or the accepting transplant center. Recently, two studies described the importance of steatosis as a donor risk factor [29,30]. Spitzer et al. [30] concluded that steatosis should be added to the DRI, when dealing with a high-risk donor. However, objective evaluation of the range of steatosis, either as macro- or microvesicular steatosis, is not standardized so far subjective to some degree [29]. We believe that routine donor biopsy evaluation may enable the design of more powerful and robust donor risk scores.

We believe that the prognostic power of the DRI and ET-DRI is further significantly undermined by the fact that graft acceptance decisions on offered organs for transplantation are not documented and evaluated in these scores. There is an urgent need to evaluate and address this shortcoming immediately, especially on the background of the current liver transplant scandals in Germany [23,31]. We believe that this aspect is a relevant issue in liver transplantation today. Transplant centers regard their graft acceptance policy as their potential advantage in a highly competitive area for a very scarce resource in times of ubiquitous organ shortage. It can be assumed that these aspects of potentially unwanted transparency by the proponents of transplantation stood in the way of previous attempts to generate robust prognostic scores that include donor organ quality. This may represent a deeper reason for the notorious difficulty to assess donor organ quality and its impact on prognosis after transplantation objectively.

The balance of equity and utility in donor organ allocation is a serious ethical dilemma. Several risk prediction models are available and their head to head comparisons would benefit from standardized reporting and formal, consistent statistical evaluation [18,26]. It was claimed previously and in a different context that outcome selection and optimism biases may affect literature on prognostic scores in medicine [26].

We would like to propose joint efforts to develop a robust and objective donor risk score in an international collaboration that should include the available large data-sets from multiple institutions. We believe that the time has come for a consensus on the reporting of the reasons for declined organ offers and on quality standards for prognostic scores and their statistical analysis and validation. Jacob et al. [18] proposed a reasonable scientific approach to the latter. High quality scientific journals should make uniform requirements mandatory for the reporting of prognostic scores.

## Conclusion

The results of this current study clearly show that the ET-DRI is not applicable in the population from our center within the Eurotransplant community for the prediction of patient or graft survival. On the background of a paucity of proper prognostic models, justified organ allocation which weighs urgency against prediction of success of liver transplantation must be the aim of future research in this field.

## Patients and methods

### Ethics statement

As an observational retrospective study, according to the *Professional Code* of the German Medical Association (article B.III. § 15.1), neither informed consent nor approval of the ethics committee was needed for this study.

### Patients

291 consecutive liver transplants were included to analyze the model’s accuracy and applicability for this dataset of a major transplant centre in the Eurotransplant community. Included were 20 (6.9%) split liver transplants, 30 (10.3%) acute re-transplants (retransplantation within 30 days after the previous transplant) and 25 (8.6%) chronic retransplants (primary transplantation n = 235, secondary transplantation n = 48, tertiary transplantation n = 6, quaternary transplantation n = 1) in a total of 257 patients (median age: 49.6 years, range: 18–69 years; males n = 177 (64.4%), females n = 114 (35.6%), ratio males/females: 1.8). All transplants were performed in our centre between the 01/01/2007 and the 12/31/2010. The post-transplant observational period ended on the 12/31/2011. Indications leading to liver transplantation as well as the most probable leading causes of death in our cohort are summarized in Tables 3 and 4.

**Table 3:** Shown are the indications for liver transplantation in the study population

**Indications for liver transplantation:**	**%**
acute liver failure	10.3
alcoholic cirrhosis	8.6
alpha-1-antitrypsin deficiency	1.4
autoimmune hepatitis	1.0
biliary atresia	0.7
Budd Chiari syndrome	2.1
cryptogenic cirrhosis	5.5
familial amyloidotic polyneuropathy	1.0
HBV HCV related cirrhosis	0.3
HBV related cirrhosis	4.5
HCC	19.6
HCV related cirrhosis	5.5
intrahepatic CCC	0.7
M. Osler/hemangioma	0.3
neuroendocrine metastases	1.0
Other	0.7
Oxalosis	0.3
PBC	2.7
polycystic disease	3.4
PSC	8.2
re-TX: biliary complications	3.4
re-TX: chronic graft failure	5.5
re-TX: chronic rejection	1.0
re-TX: primary graft non-function	6.2
re-TX: recurrent viral hepatitis	0.3
re-TX: vascular complications	2.4
secondary biliary cirrhosis	1.7
Wilson disease	1.4
**Total:**	100.0

(HBV = hepatitis b; HCV = hepatitis c; CCC = cholangiocellular carcinoma, PBC = primary biliary cirrhosis, PSC = primary sclerosing cholangitis, re-Tx = retransplantation).

**Table 4:** Shown are the most probable causes of death in our study population

** Most probable causes of death:**	**%**
cardiovascular event	12.2
cerebral: bleeding	2.1
cerebral: ischemia	4.7
data not available	10.5
de novo malignancy	3.2
gastrointestinal: ischemia	3.2
gastrointestinal: perforation	2.1
Infection: fungal	5.8
Infection: sepsis	21.1
intraabdominal bleeding	4.7
liver graft: biliary tract complications	12.2
liver graft: initial graft non-function	2.1
lung: ARDS	8.5
lung: pneumonia	3.2
polytrauma	0.6
social: suicide	0.6
tumor recurrence	3.2
**Total deaths:**	**100.0**

(HBV = hepatitis B; HCV = hepatitis C; ARDS = Acute Respiratory Distress Syndrome).

### Study end-points

Primary study endpoints were 3-month and 1-year patient mortality and 3-month and 1-year graft survival. (Graft survival and failure free survival (FFS) are synonyms that describe the time period from date of transplantation until the date of retransplantation or recipient death).

### Statistical analysis

For the primary study endpoints ROC-curve analysis was performed to calculate the sensitivity, specificity, and overall model correctness of the ET-DRI as a predictive model. AUROCS larger than 0.7 indicate a clinically useful prognostic model [18-22]. The respective cut-off values of the potential prognostic models were determined with the best Youden index (Youden index = sensitivity + specificity – 1) [24]. If possible, the Hosmer-Lemeshow Chi^2^-statistic goodness-of-fit test was applied to assess model calibration of the binary logistic regression models. These statistics suggest good fit when the associated p-values are greater than 0.05 [21,25]. Survival analysis was performed with the Kaplan-Meier method. The statistical influence of the determined cut-off values on patient and graft survival was analyzed with the Log Rank test. Binary logistic regression was performed as additional test to determine the influence of the ET-DRI on study end-points. For all statistical tests a p-value <0.05 was defined as significant. The SPSS statistics software version 20.0 (IBM, Somers, NY, USA) was used to perform statistical analysis.

### Calculation of the ET-DRI

The ET-DRI was calculated as described before with this formula [5]:

ET-DRI = exp[0.960((0.154 if 40 ≤ age < 50) + (0.274If 50 ≤ age < 60) + (0.424 if 60 ≤ age < 70) + (0.501 if 70 ≤ age) + (0.079 if COD = anoxia) + (0.145 if COD = cerebrovascular accident) + (0.184 if COD = other) + (0.411 if DCD) + (0.422 if partial/split) + (0.105 if regional share) + (0.244 if national share)) + (0.010 × (cold ischemia time − 8 h)) +0.06((latest lab GGT (U/L)- 50)/100) + (0.180 if rescue offer)].

Regional share was defined by Braat et al. as allocation within Germany and national share as allocation within Eurotransplant land [5].

### Data collection

This is a single centre analysis complemented by ongoing data collection.

### Setting

The setting of the study is a university hospital in Germany within the Eurotransplant community.

### Inclusion and exclusion criteria

Included were all consecutive liver transplants performed in adult recipients (minimum age 18 years). Excluded were all combined transplants (e.g. combined liver and kidney transplantation) and living-related organ donor transplants.

## Abbreviations

DRI: Donor risk index; ET-DRI: Eurotransplant-donor risk index; AUROC: Area under the receiver operating characteristic; MELD: Model of end-stage liver disease; ECD: Extended donor criteria.

## Competing interests

The authors have no competing interests to declare. They did not receive any funding for this work.

## Authors’ contribution

BR, AK and HS designed the study and accumulated the data. BR, AK, AG and HS analyzed and interpreted the data and drafted the manuscript. All authors read and approved the final manuscript.
